# Caring under pressure: economic social determinants of health influence family caregivers

**DOI:** 10.3389/fpubh.2025.1676656

**Published:** 2025-11-19

**Authors:** Payton Radcliffe, S. Alison Bolling, Julie Hicks Patrick

**Affiliations:** Department of Psychology, West Virginia University, Morgantown, WV, United States

**Keywords:** caregiving, behavioral risk factor surveillance system, stress, well-being, social determinants of health

## Abstract

Health disparities, the unequal onset, severity, and treatment of chronic health conditions, are differentially experienced by adults living in different geographic locations in the United States. Coupled with an earlier onset and more severe symptomatology among patients, family care partners may also experience increased stress related to other place-based disparities, including limited access to care and increased economic challenges. Using data from the 2023 Centers for Disease Control and Prevention’s Behavioral Risk Factor Surveillance System, we examine the influences of caregiver resources, caregiving demands, and place-based factors in a structural equation model aimed at understanding caregiver physical and emotional health. Data from 5,432 family caregivers (Mean age ~ 66 yrs.; ~60% female; 10% rural) who provide a range of assistance with personal care tasks (49%) and household tasks (78%) were used in a multigroup analysis examining the unique contributions of rurality to this model of caregiver stress. Although rurality does not differentially increase psychological wellbeing challenges among caregivers, rural caregivers did experience exacerbation of physical health challenges when compared to their urban counterparts. *Post hoc* analyses are conducted in order to isolate this effect and to inform policy and program recommendations.

## Introduction

Relative to their non-caregiving counterparts, family caregivers may experience lower emotional wellbeing, which is often attributed to the stresses of providing care. Fifty years of caregiving research have documented the emotional, physical, social, financial, and space constraints that exacerbate caregiving stresses (1). Building on these important foundations, more recent work has begun to examine the influence of pre-caregiving stressors on families (2). Other work takes a more nuanced approach to identify the specific aspects of caregiving that might give rise to threats to caregiver wellbeing (3). The purpose of the current study is to examine the potential effects of caregiving stressors within a broader framework of stressors that include one’s economic context.

### General stress as the linchpin in the caregiving-wellbeing nexus

Broadly defined, stress is a process whereby stimuli evoke a physiological and/or emotional reaction (4, 5). These responses, particularly in the face of ongoing and chronic exposure, may result in decreased physical and emotional wellbeing. Although researchers and clinicians may ask domain-specific questions about experienced stress, it is unclear whether most adults are able to decompose their experienced stress into its constituent parts. For example, Luo and colleagues (6) recently used two samples from the Health and Retirement Study to examine the factor structure of general versus domain-specific stress. They focused on the domains of family/interpersonal stress, job-related stress, racial discrimination, and neighborhood changes. General stress at time 1 predicted health at time 2 (4 years later), with only the neighborhood domain emerging as a unique domain-specific predictor. Thus, although adults are able to identify sources of stress across domains, it is the general stress factor which seems to underly physical health outcomes. Of note, individual characteristics were associated with higher perceived general stress, including younger ages, female sex, non-white racial identity, higher neuroticism, and more recent exposure to stressors. These same characteristics are often associated with family caregivers.

Although more than half of caregivers report experiencing stress while providing care (7), it is unclear whether this relates to an individual propensity for experiencing stress, the nature of the care tasks, or other environmental demands. Data from the HRS (2) were used to investigate pre-caregiving and post-caregiving trajectories among 4,812 adults who reported the physical and cognitive decline of their mother. Results suggested that it was not caregiving, per se, that was associated with increased depression among these adult caregivers, but rather, being exposed to the health declines of a family member served as a salient stressor. Among these adults providing care to their aging mothers, dementia and functional disability was especially challenging and these challenges increased threats to wellbeing over time among the caregiving adult children (2). Similarly, recent work by Smith et al. (3) identified aspects of caregiving that resulted in increased threats to emotional wellbeing. Whereas care receiver characteristics were associated with demands on caregiver time, the difficulty of performing those care tasks was associated with increased depression. Thus, a more nuanced approach to understanding pre-existing patterns and resources, as well as current care demands, may be informative.

### eSDOH and family caregiving

Among non-caregivers and caregivers alike, there are multiple sources of stress in an adult’s life, including economic challenges and other Social Determinants of Health (SDOH) (8). SDOH include environmental conditions that affect one’s health and quality of life, such as where people work, live, and age. Other non-demographic SDOH incude access to health care, education, food, and preventative health behaviors (9). Economic social determinants of health (eSDOH), may include loss of employment, difficulty with finances, and difficulty with transportation. That such financial strain is associated with increased depression is not surprising (10).

Indeed, the effects of financial strain on caregivers have been studied for decades. However, such challenges have not been examined using SDOH frameworks. It is clear that when the caregiving context requires, many caregivers reduce their paid employment to accommodate (11), but such options are not available to all families. The strains that financial stress may add to caregiving have not been adequately studied.

The current study focuses on the contributions of the caregiving context and specific eSDOH to experienced stress and emotional wellbeing. Caregivers face many stressors. Disentangling the sources of the stress, including those experienced by non-caregivers, has the potential to provide a better understanding of wellbeing and to potentially guide interventions. Thus, we sought to examine whether the effects of caregiving and eSDOH differentially influence experienced stress and ultimately wellbeing.

## Methods

### Data source

Data for these analyses were provided by adults who completed the 2023 Behavioral Risk Factor Surveillance System (BRFSS), released by the Centers for Disease Control and Prevention in September 2024 (12). The BRFSS is a national interview administered annually to assess a variety of health risks, health behaviors and general health status among adults ages 18 + living in the community in all 50 US states and three US territories. More than 420,000 adults participated in the 2023 survey. All participants were asked: “During the past 30 days, did you provide regular care or assistance to a friend or family member who has a health problem or disability?” (12). A total of 11,502 responded in the affirmative (22.6%). The 2023 BRFSS included an optional module about economic social determinants of health (eSDOH) (12). About half of the caregivers answered these items. Thus, the following analyses are based on responses from 5,432 caregivers who provided answers to all of the items of interest in the current study. Finally, we note that we acquired the data in September 2024. Some items (i.e., eSDOH) in our analyses may no longer be available to the public.

### Participants

Among the 5,432 caregivers in the current sample, the average age was 57.5 years (SD = 16.4; range 18 to 80+). Approximately 40% identified as male. The optional caregiving module was offered by only five states in the 2023 BRFSS. Specifically, the module was offered in: Arizona, Arkansas, Hawaii, Idaho, and Louisiana. Approximately 11.4% of the current sample resided in rural areas. The analytic sample was less racially diverse than the US as a whole (13). Within the current sample, 66.7% identified as white non-Hispanic (64%), 6.5% identified as Black non-Hispanic (12%; US Census), and 8.8% identified as Hispanic (16%) (13). Additionally, 11.1% of the current sample identified as a member of another non-Hispanic group, and 6.9% identified as multiracial. The median annual household income was in the $25,000 to $35,000 range. More than half (61.2%) were married or part of a long-term couple, but 2.3% were separated from their spouse, 13.1% were divorced, 8.5% were widowed, and 14.4% were single, never married. The sample was well-educated, with 95.2% having earned a high school diploma or equivalent. About 40% had earned a 4-year college degree.

Among the caregivers, 1,535 (28.3%) were providing care to parents or parents-in-law. An additional 1,302 were caring for a spouse or partner (24%). A third group included 1,578 adults (29.1%) who were providing care to another relative, including a child (*n* = 565), grandchild (*n* = 54), sibling (*n* = 434), grandparent (*n* = 185), and other relative (*n* = 358). A fourth group of 1,017 (18.7%) were providing care to a friend. Reasons prompting the need for care varied, but included physical health conditions (23.2%) such as kidney or lung diseases, cancer (8.4%), developmental disabilities such as Down Syndrome (5.5%), emotional illness and substance abuse (4.7%), injuries like broken bones (7.2%), “old age” or general frailty (16.5%), and other conditions (34.4%).

### Measures

**Figure 1:**
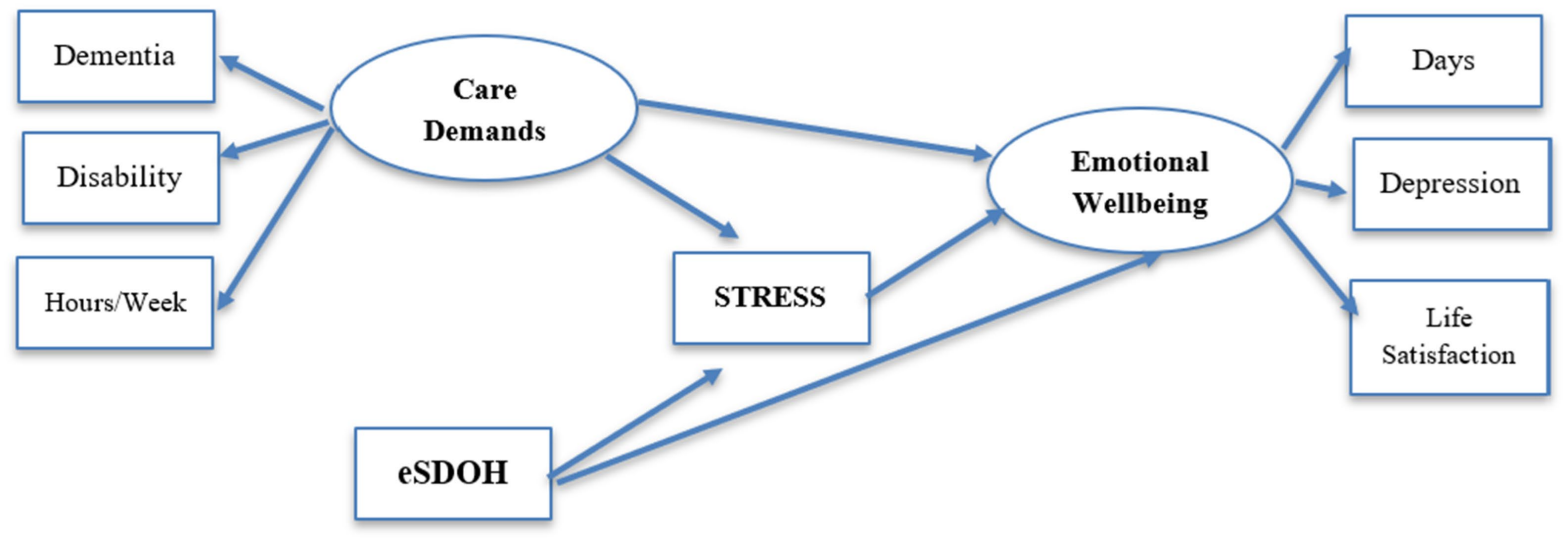
Conceptual model.

To test the model depicted in Figure 1, we examined indices of the caregiving context, economic SDOH, stress, and emotional wellbeing. Each is described below.

#### Caregiving context

Caregiving context was assessed using three indicators. Caregivers were asked whether the care recipient had dementia or other cognitive impairment, to which 16.7% replied in the affirmative. Functional ability of the care recipient was indexed by the sum of two items, which queried whether the caregiver assisted with personal care tasks (49.3%) or household tasks (79.7%). The two dichotomous items were summed, and the mean was used in subsequent analyses (*M* = 1.29; SD = 0.72). A third index was formed based on the number of hours per week caregivers assisted. The categorical responses were recoded to the midpoint, and included less than or equal to 8 h (53.9%, recoded to 4 h), 9 to 19 h per week (14.0%, recoded to 14), 20 to 39 h per week (12.0%, recoded to 30), and 40 or more hours per week (20.1% recoded to 40).

#### eSDOH

Participants reported whether during the past year, they received food stamps (11.3%), were unable to pay their bills (12.6%), had their utilities disconnected for failure to pay (8.9%), had their work hours reduced or were terminated from their job (11.8%), and whether lack of reliable transportation was a problem (9.3%). Although most (69.7%) reported none of these economic SDOH, 16.8% experienced one and 13.6% reported two or more. Thus, despite the range being 0 to 5 (*M* = 0.54, SD = 1.0, *α* = 0.66), the count measure operates as a dichotomous variable.

#### Daily stress

Daily stress was assessed using a single item, “Stress means a situation in which a person feels tense, restless, nervous, or anxious, or is unable to sleep at night because his/her mind is troubled all the time. Within the last 30 days, how often have you felt this kind of stress?” Most reported never (29.6%) or rarely (28.0%) feeling such stress. However, 25.4% reported sometimes, 9.2% reported usually, and 7.8% reported always feeling such pervasive stress. Responses were recoded such that higher scores reflected more frequent experiences of stress, resulting in a sample mean of 2.38 (SD = 1.22).

#### Caregiver wellbeing

Caregiver wellbeing was indexed using three items. Caregivers were asked the number of days in the past 30 days during which their mental health was poor. Including the 53% who responded that they had zero poor mental health days, the sample mean number of poor mental health days was 5.71 (SD = 9.4; range 0 to 30). Exploratory analyses examined whether a log transformation was needed, given the potential for zero-inflation. Results with the raw and transformed index were essentially the same, so we report raw scores herein. Caregivers also reported whether they had ever been diagnosed with depression, a depressive disorder, or anxiety, with 26% responding in the affirmative. Finally, participants were asked, “In general, how satisfied are you with your life?” Higher scores reflect more dissatisfaction, such that 1.4% were very dissatisfied, 4.6% were dissatisfied, 51.1% were satisfied, and 42.9% were very satisfied. The item mean was 1.64 (SD = 0.64).

## Analyses

### Preliminary analyses

Bivariate associations for the sample were examined using Pearson correlation coefficients for continuous variables and Spearman correlations for categorical variables. Due to the large sample used in the current study, smaller magnitude correlations reached statistical significance. Thus, we focus on those coefficients with magnitudes greater than 0.20 and *p*-values smaller than 0.001. Coefficients are presented in Table 1.

**Table 1:** Descriptive statistics and correlations for study variables.

	1	2	3	4	5	6	7	8	9	10	11
1. Age	—										
2. Sex †	0.00	—									
3. Relationship †	0.13**	0.02	—								
4. MH days	−0.25**	−0.09**	0.01	—							
5. Satisfaction with life	−0.14**	0.00	−0.011	0.37**	—						
6. Depression †	−0.18**	0.12**	−0.01	0.46**	−23**	—					
7. Stress	−0.29**	0.12**	−0.033*	0.52**	0.39**	0.39**	—				
8. eSDOH	−0.31**	0.03**	0.03*	0.32**	0.29**	0.25**	0.27**	—			
9. CR Disability	−0.05**	−0.07**	0.05**	0.08**	0.06**	0.09**	0.09**	0.10**	—		
10. CR Dementia †	0.03*	0.04**	−0.04**	0.04**	0.04**	0.05**	0.06**	0.01**	0.12**	—	
11. Hours/week	−0.09**	−0.02	0.07**	0.07**	0.07**	−0.45**	0.11**	0.10**	0.84**	−0.12**	—
Mean	57.13	1.60	2.38	5.71	1.64	0.26	2.38	0.54	0.49	0.17	1.29
Standard deviation	16.37	0.49	1.08	9.45	0.64	0.44	1.22	1.00	0.50	0.38	0.72

† Spearman’s ρ used.

*p < 0.05, **p < 0.01.

The model represented in Figure 1 was tested in AMOS v. 29.0.0. AMOS uses maximum likelihood procedures to simultaneously estimate measurement and structural paths in the model. Individual paths are tested for statistical significance using the Critical Ratio (CR), for which values greater than 2.16 are significant at *p* < 0.01 and CR > 3.29 are significant at *p* < 0.001. Because the chi-squared statistic is sensitive to small deviations between the tested and inherent models in larger samples, we relied on additional indices of fit. We used the comparative fit index (CFI), for which values greater than 0.90 suggest an acceptable fit, but values greater than 0.95 being preferred. We examined the Tucker-Lewis Index (TLI), for which values greater than 0.90 indicate an acceptable fit. We also used the root mean square error of approximation (RMSEA), for which values less than 0.08 are considered acceptable, and values less than 0.05 indicate a close fit of the model to the data (14, 15).

As shown in the upper portion of Table 2, the three indices of Wellbeing loaded well onto the latent construct. The lower portion of the table displays the individual regression paths. Results of the analysis suggest a close fit of the model to the data, *X*^2^ (DF = 17; *N* = 5,432) = 168.09, *p* < 0.001; CFI = 0.977; TLI = 0.961; RMSEA = 0.040. The model accounted for 55.6% of the variance in wellbeing and 13.1% of the variance in stress. When inspecting individual regression paths, it is important to remember that due to the large sample size of the current study, relatively small beta weights resulted in statistically significant effects. All regression paths were significant.

**Table 2:** Results of SEM testing (*N* = 5,432).

	*b*	β	S. E.	C. R.	*p*
Measurement model					
Poor mental health days←Wellbeing	1.0	0.732			
Life Satisfaction←Wellbeing	0.048	0.517	0.002	31.30	***
Depression←Wellbeing	0.036	0.559	0.001	33.44	***
Dementia←Care Demands	1.0	0.155			***
Functional Disability←Care Demands	8.73	0.701	1.20	7.29	***
Hours per Week←Care Demands	145.63	0.571	17.71	8.23	***
Structural model					
Wellbeing←Stress	3.566	0.629	0.089	40.03	***
Stress←Care Demands	2.622	0.125	0.453	5.79	***
Wellbeing←Care Demands	3.608	0.030	2.098	1.72	0.085
Stress←eSDOH	0.410	0.340	0.015	26.81	***
Wellbeing←eSDOH	1.591	0.233	0.098	16.28	***

*X*^2^ (DF = 17; *N* = 5,432) = 168.09, *p* < 0.001; CFI = 0.977; TLI = 0.961; RMSEA = 0.040.

R^2^ Stress = 0.131; R^2^ Wellbeing = 0.556.

***p < 0.001.

As shown in Table 2, poorer wellbeing was associated with more stress (*β* = 0.63, *p* < 0.001) and more eSDOH (*β* = 0.23, *p* < 0.001), but not directly linked with caregiving demands (*β* = 0.03, *p* = 0.085). Both caregiving demands (*β* = 0.13, *p* < 0.001) and eSDOH (*β* = 34, *p* < 0.001) directly contributed to stress. We examined the indirect effects of caregiving demands and eSDOH on wellbeing through their association with stress. In both cases, stress mediated the effects on wellbeing. The specific indirect effect of caregiving demands on wellbeing through stress (*β* = 0.079) was significant (bias-corrected 95% CI = [0.064, 0.105], *p* = 0.004). Similarly, the indirect effect of eSDOH on wellbeing via stress (*β* = 0.214) was significant (bias-corrected 95% CI = [0.198, 0.231], *p* = 0.007). We note that the CI do not overlap, with a stronger effect of eSDOH on wellbeing than caregiving demands on wellbeing.

### Moderation testing

In order to examine whether the relationship to the care recipient altered the associations within the model, we conducted a multigroup moderation analysis comparing model functioning across caregivers to parents, spouses, other relatives, and friends. Table 3 presents standardized direct and indirect effects for these four groups of caregivers. As shown in Table 3, only small differences in the strength of associations were observed across the four groups.

**Table 3:** Relationship to care receiver moderation analyses.

Betas and CI	Care-receiver is:			
	Parent (*n* = 1,535)	Spouse (*n* = 1,302)	Other family (*n*= 1,578)	Friend (*n* = 1,017)
Standardized direct effects (β)				
Wellbeing←Stress	−0.643***	−0.605***	−0.670***	−0.567***
Stress←Care Demands	−0.080**	−0.142***	−0.140***	−0.093*
Wellbeing←Care Demands	−0.039 ns	0.020 ns	0.074*	0.055 ns
Stress←eSDOH	−0.343***	−0.330***	−0.298***	−0.410***
Wellbeing←eSDOH	0.240***	0.269***	0.168***	0.294***
*X*^2^ (DF = 68) = 299.09, *p* < 0.001; CFI = 0.964; TLI = 0.941; RMSEA = 0.025				
R^2^ Wellbeing	0.549	0.564	0.564	0.554
R^2^ Stress	0.129	0.109	0.109	0.176
Standardized indirect effects (β)				
Care Demands→Stress→Wellbeing	0.051**	0.086**	0.094**	0.052**
eSDOH→Stress→Wellbeing	0.221*	0.199*	0.200*	0.232*

*p < 0.05, **p < 0.01, ***p < 0.001.

## Discussion

Decades of research show that family caregivers often experience struggles in caring for their family members (1). More recent studies expand attention to include the broader context in which family caregiving occurs. To that end, we examined the influences of caregiving demands and economic demands on stress and wellbeing among caregivers. Using data from the 2023 CDC, we are able to assess these effects in a large sample of adults.

By including both caregiving demands and economic SDOH in a single model, we are able to directly assess their associations with both experienced daily stress and longer-term emotional wellbeing. Moreover, we are able to compare their direct effects on wellbeing and their indirect effects on wellbeing via experienced stress. Results of our omnibus structural model were profound: although caregiving demands exert indirect effects on wellbeing through their association with experienced stress, caregiving demands, per se, did not directly result in threats to wellbeing. In contrast, economic SDOH exerted strong direct and indirect effects on wellbeing. When comparing the standardized beta weights, the effect for eSDOH was nearly three times that of caregiving demands. This finding is particularly disturbing because the measure of eSDOH is somewhat crude and was operating much like a dichotomous variable. Adults who expressed nearly any difficulty paying their bills, had their utilities disconnected, were challenged to obtain sufficient food, or did not have reliable transportation were at an especially high risk for threats to their wellbeing. Caregiving clearly adds to stress and likely exacerbates the effects of eSDOH on wellbeing.

Thus, although consistent with the general stress factor identified in the HRS data (6), our results extend beyond the identification of a pervasive stress factor. Given the strong effects of economic burdens, social safety nets like Medicaid and other sources may become critical for supporting families who are providing care amidst other financial challenges. Moreover, although we had thought that the strength of predictors within the model might vary as a function of different relationships to the care-receiver, that was not the case. Across relationships, having difficulty paying one’s bills, experiencing food insecurity, and lacking reliable transportation was associated with more stress and ultimately, poorer emotional wellbeing. These results fit well with the nuanced investigation by Han (2), suggesting that experienced stresses among caregivers is exacerbated by specific contextual influences. For Han (2), dementia and functional disability in the care receiver were the primary stressors. Our analyses suggest that caregiving research must expand to include broader contexts, including economic challenges.

### Strengths, limitations, and implications

This is a novel and critical study that brings forth a more accurate understanding of informal caregiving within the context in which it occurs. Recognizing that experienced stress has a variety of potential causes, and thus, interventions may help us to better support family caregivers.

Although prior work has acknowledged that caregiving may affect several domains, including finances and employment (1), and it is reasonable to assume that poor emotional wellbeing outcomes among caregivers are associated with the care they provide, our analyses suggest a more holistic view is appropriate. Specifically, caregiving tasks added to experienced stress, but they did not directly affect wellbeing. However, the daily financial hardships that families were experiencing did exert both direct and indirect effects on wellbeing. This finding suggests that public health may be well-served by broader financial safety nets for caregivers and non-caregivers alike. Moreover, alleviating such daily sources of stress might result in improved care-receiver outcomes, as well. Finally, reciprocal effects between stress and wellbeing across different contexts must be examined. However, these hypotheses are best tested using more sophisticated caregiving data that includes longitudinal examinations.

We examined the experiences of caregiving adults using public access health surveillance data from the CDC’s BRFSS. Thus, as a secondary data set, we did not control the breadth or depth of caregiving items posed. In addition, the caregiving module is an optional module in the BRFSS. As such, only five states included it in 2023. It is likely that local resources in Arizona, Arkansas, Hawaii, Idaho, and Louisiana differ from other areas in the United States. Geographic differences are likely to be key factors shaping the relation among economic SDOH, stress and other stressors. However, the caregiving module was selectively launched by states and the 2023 BRFSS does not allow a rigorous examination of these regional differences in caregiving experiences. Similarly, because it is a surveillance survey, the BRFSS module includes only a handful of items. For example, although the number of hours per week and tenure of caregiving are queried, only three ADL/IADL tasks are included. Other items that could inform this research, such as emotional closeness and specific caregiving burden, are not included. These data also do not provide information about whether caregiver status is voluntary or whether there are other caregivers who support the car receiver.

Despite these limitations, our study adds to the literature on family caregiving. Specifically, our study is among the first to examine caregiving demands within the context of other daily stressors. By situating caregiving in this way, we are able to begin to disentangle the effects of economic stresses and caregiving stresses on overall emotional wellbeing. Therefore, it would be helpful to conduct a longitudinal study with interventions included and ask more focused caregiving questions. This can allow us to pinpoint caregiver stress changes over time and identify variables and interventions that may influence those changes in a positive way.

## Conclusion

The significant direct influences of experienced stress and eSDOH on caregivers’ wellbeing reinforce the need for public health intervention programs to support caregivers and those who are struggling with eSDOH threats.

## Data Availability

Publicly available datasets were analyzed in this study. These data can be found here: BRFSS 2023 data are available: https://www.cdc.gov/brfss/annual_data/annual_2023.html.
